# Vitamin E-induced coagulopathy in a young patient: a case report

**DOI:** 10.1186/s13256-023-03827-y

**Published:** 2023-03-23

**Authors:** Ritika Abrol, Reshma Kaushik, Deepak Goel, Sonu Sama, Rajeev Mohan Kaushik, Mansi Kala

**Affiliations:** 1 Department of General Medicine, Himalayan Institute of Medical Sciences, Swami Rama Himalayan University, Jolly Grant, Dehradun, Uttarakhand 248016 India; 2 Department of Neurology, Himalayan Institute of Medical Sciences, Swami Rama Himalayan University, Jolly Grant, Dehradun, Uttarakhand 248016 India; 3 Department of Critical Care Medicine, Himalayan Institute of Medical Sciences, Swami Rama Himalayan University, Jolly Grant, Dehradun, Uttarakhand 248016 India; 4 Department of Pathology, Himalayan Institute of Medical Sciences, Swami Rama Himalayan University, Jolly Grant, Dehradun, Uttarakhand 248016 India

**Keywords:** Acquired coagulation factor deficiency, Coagulopathy, Vitamin E, Vitamin E toxicity

## Abstract

**Background:**

High-dose vitamin E intake is known to inhibit vitamin K-derived coagulation factor synthesis, which can cause serious bleeding events such as gastrointestinal bleeding and intracranial hemorrhage. We report a case of coagulopathy induced by marginally increased levels of vitamin E.

**Case presentation:**

A 31-year-old Indian man presented with oral bleeding, black tarry stools, and bruising over his back. He had been taking non-steroidal anti-inflammatory drugs for low backache and vitamin E for hair loss. He had mild anemia with normal platelet count, thrombin time, and prolonged bleeding time, activated partial thromboplastin time, and prothrombin time. Serum fibrinogen was slightly raised. Mixing studies with pooled normal plasma, aged plasma, and adsorbed plasma were suggestive of deficiency of multiple coagulation factors due to acquired vitamin K deficiency. Serum phylloquinone was normal, while prothrombin induced by vitamin K absence-II level was increased. Serum alpha-tocopherol was slightly raised. Upper gastrointestinal endoscopy showed multiple gastroduodenal erosions. A final diagnosis of vitamin E toxicity-related coagulopathy was made. The patient responded well to pantoprazole, vitamin K supplementation, multiple fresh frozen plasma transfusions, and other supportive treatments besides the discontinuation of vitamin E supplementation. The coagulation parameters normalized, and the patient was discharged with complete resolution of symptoms and remained asymptomatic during the follow-up for 6 months.

**Conclusions:**

Vitamin E-related inhibition of vitamin K-dependent factors with coagulopathy may occur even at marginally increased levels of serum vitamin E. This risk becomes significant in patients receiving other drugs that may increase the risk of bleeding.

## Introduction

Vitamin E toxicity is rarely observed due to its ready excretion in bile and urine [1]. However, it has been noted to occur in cases of lipid or hepatobiliary disorders where its absorption or excretion may be altered. High-dose vitamin E supplementation is known to inhibit vitamin K-related enzyme activation relevant for coagulation factor syntheses, such as factors II, VII, IX, and X, as well as protein C and S. Deficiency of factors II, VII, IX, and X promotes bleeding [2], while deficiency of protein C and protein S leads to the loss of their natural anticoagulant effects, with consequent unrestrained thrombin generation, resulting in thromboembolism [3, 4]. Generally, deficiency of factors II, VII, IX, and X is the dominant effect and may lead to bleeding. The mechanism of inhibition of vitamin K-related enzyme activation relevant for coagulation factor synthesis is evidenced by the elevation of levels of prothrombin induced by vitamin K absence-II (PIVKA-II), due to an increase in under-gamma-carboxylated prothrombin.

Vitamin E excess further inhibits factor IX activation by reducing vitamin K-dependent carboxylation of glutamate and inhibits platelet aggregation [5]. Thus vitamin E toxicity can present as coagulopathies alongside general complaints such as malaise, nausea, myalgia, and fatigue [6].

Though it is generally understood that vitamin E toxicity may occur if consumed at levels greater than 1000 mg/day, there is no fixed cut-off point [7]. The circulating alpha-tocopherol levels depend heavily on the lipid content of the blood. Vitamin E cannot be measured accurately by circulating alpha-tocopherol levels in patients who have very high or very low cholesterol levels. The same applies to patients with average cholesterol levels as well. This is because of the upregulation of biliary and urinary excretion once vitamin E levels increase in the body [5]. Because of these irregularities in vitamin E metabolism, the minimum dose, form, and duration of vitamin E intake required to induce a clinically significant effect on coagulation pathways are not known [7].

Vitamin E toxicity may be overlooked as the cause of coagulopathy if serum levels of vitamin E are only marginally increased. We report a case of coagulopathy induced by marginally increased levels of serum vitamin E.

## Case report

A 31-year-old Indian man, a restaurateur by profession, presented to the emergency services with complaints of low backache for 1 month and oral bleeding, the passage of black tarry stools, and bruising over his back for the last seven days. He had intense nausea and fatigue for several days and began to develop bruising about 1 week ago. He experienced oral bleeding and passed black tarry stools three times, following which he decided to visit the emergency services.

The backache was non-specific in nature. The patient had been taking over-the-counter non-steroidal anti-inflammatory drugs (NSAIDs) in the form of ibuprofen 400 mg infrequently for the past 1 month for his backache, before his current symptoms of bleeding.

The patient had no complaints of bleeding tendencies in the past, such as frequent nosebleeds, prolonged bleeding from gums or injuries, gastrointestinal bleeds, bleeding into joints, easy bruising, and hematuria. There was no history of jaundice, peptic ulcer, malignancy, or bony aches and pains. He had never received any blood transfusions. There was no history of the use of anticoagulants, antiplatelet drugs, and corticosteroids. There was no history of heavy exposure to pesticides, injury, or surgery.

The patient also reported the use of vitamin E supplementation for his complaints of recent onset hair loss and dryness of the skin. He had taken vitamin E 400 mg twice daily for a period of 2 months, which was accompanied by a fairly poor diet due to work-related stressors.

He was a non-smoker. He drank alcohol sparingly and had no drug addictions. He consumed a mixed diet. He was residing in a semiurban area that had moderate air pollution levels.

He was a bachelor and had two elder brothers. His parents and brothers were healthy. There was no family history of any bleeding disorder in either parents, maternal grandparents, uncles, aunts, or siblings. There was no history of any consanguineous marriage in the family.

**Fig. 1:**
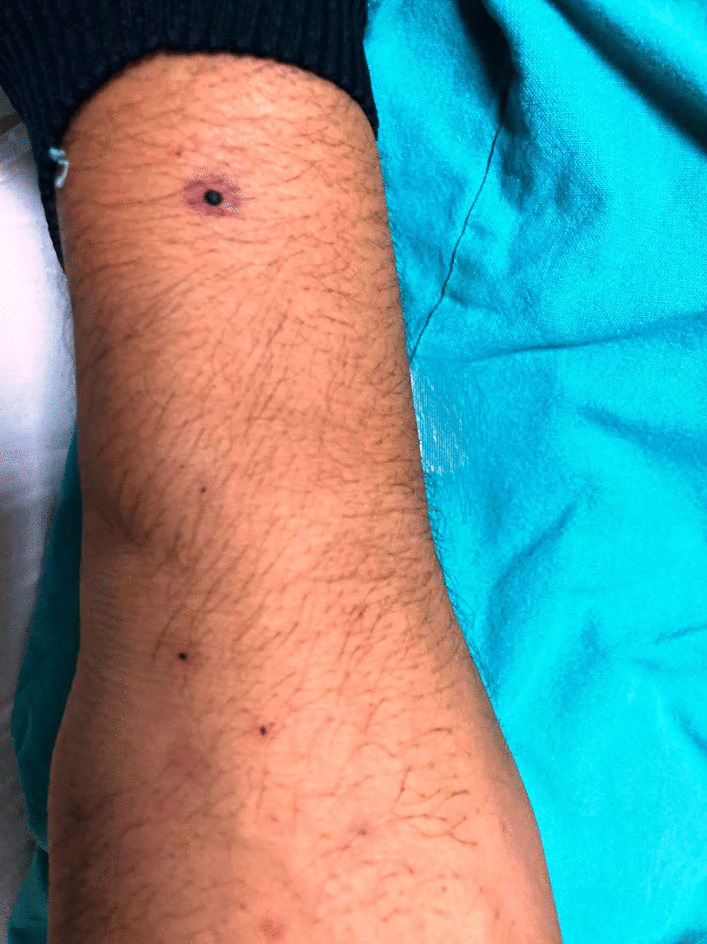
Petechiae over the arm

**Fig. 2:**
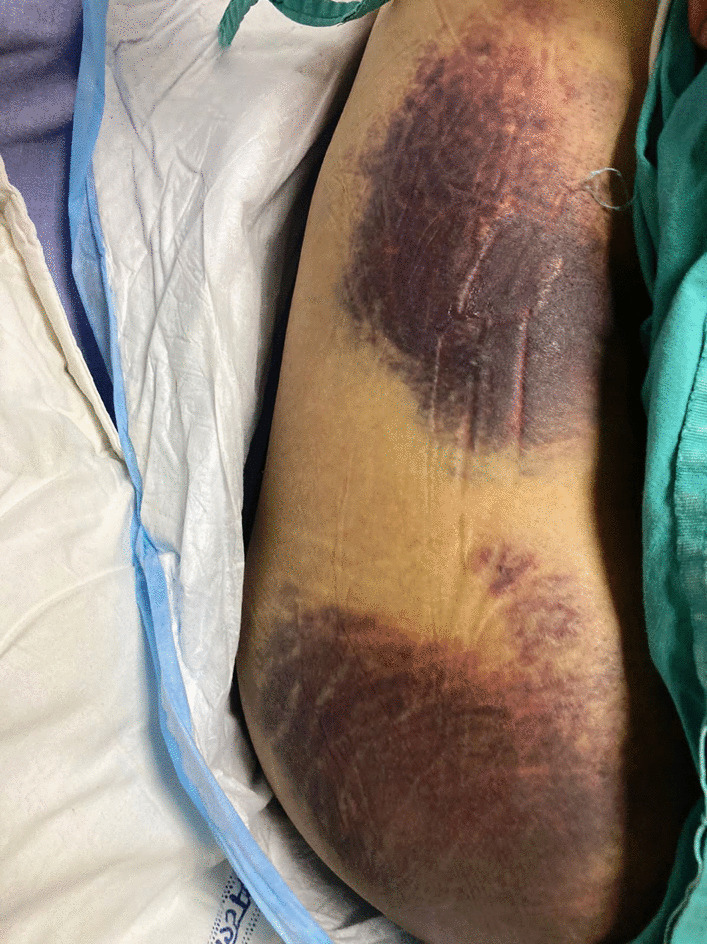
Ecchymosis over the back of the patient

On admission, the patient was hemodynamically stable. He was well-built and in no distress. His pulse rate was 74 beats per minute, blood pressure was 122/78 mmHg, and oral temperature was 37 °C. He had nonspecific thinning of hair over bitemporal regions. Mild pallor and bleeding from the gums were present. Multiple punctate, perifollicular hemorrhages were present over his forearms (Fig. 1) along with large ecchymotic patches over his back (Fig. 2). Icterus, cyanosis, clubbing, lymphadenopathy, telangiectasis, signs of chronic liver disease, and pedal edema were absent. Neurological examination showed normal higher mental functions with a mini-mental state examination (MMSE) score of 28/30. The flapping tremor was absent. The cranial nerve examination was normal. No ocular movement dysfunction or nystagmus was seen. There were no fasciculations, motor deficits, or incoordination. Tendon jerks were normal and the plantars showed bilateral flexor response. Sensations were intact.

Examination of the cardiovascular system and the respiratory system was normal. Abdominal examination showed no hepatomegaly, splenomegaly, or ascites. He was evaluated for gingival bleeding and melena.

The laboratory work-up showed low hemoglobin (11.0 g/dL) with normal mean corpuscular volume (87.2 fl), mean corpuscular hemoglobin (28.9 pg), leukocyte count (9900/mm^3^), and platelet count (2,72,000/mm^3^). Random blood sugar, liver and renal function tests, and thyroid profile were within normal range. The anemia was evaluated further. Peripheral blood film showed normocytic normochromic red blood cells, with normal white blood cells and platelets. Serum iron was 39 µg/dL, ferritin 22 µg/L, total iron binding capacity 305 µg/dL, and lactate dehydrogenase 277 U/L. Anemia was attributed to his bleeding. Bleeding time (BT) was prolonged (7 minutes, reference range 2–6 minutes). Also activated partial thromboplastin time (APTT) and prothrombin time (PT) were significantly prolonged (APTT 104.5 seconds, reference range 29–33 seconds; PT > 1 minute, reference range 11.4–14.8 seconds). Thrombin time (TT) was normal (18 seconds, reference range 14–20 seconds). Mixing studies were performed with pooled normal plasma, aged plasma, and adsorbed plasma. Full correction for APTT and PT occurred with pooled normal plasma. Mixing studies with aged plasma as well as adsorbed plasma did not show correction of abnormal PT of the patient’s plasma and were suggestive of deficiency of multiple factors. Factor VIII assay was 100% (reference range 50–150%). Factor IX assay was 9% (reference range 50–150%). The serum fibrinogen level was slightly raised (430 mg/dL, reference range 200–400 mg/dL). Due to the unavailability of resources, further factor assays were not performed.

A possibility of acquired vitamin K deficiency was considered in view of the deficiency of multiple coagulation factors. The patient was investigated further to elucidate the etiology. Serum phylloquinone (vitamin K1) level was normal (1.14 ng/mL, reference range 0.10–2.20 ng/mL). Serum PIVKA-II level was measured by chemiluminescent microparticle immunoassay (CMIA) and was increased (326.7 mAU/mL, upper normal limit 40 mAU/mL). Serum alpha-tocopherol, which is considered a surrogate marker for serum vitamin E level, was evaluated and found to be slightly raised (20.10 mg/L, reference range 5–18 mg/L).

Ultrasound examination of the abdomen was normal. Upper gastrointestinal endoscopy showed few gastroduodenal erosions with no active bleeding. Urinalysis did not show any abnormality. The stool was negative for occult blood.

Chest X-Ray and X-Rays of the lumbosacral spine were normal. The patient was further evaluated for ankylosing spondylitis, in view of the history of backache, to identify the association of any collagen-vascular disease with his bleeding complaint, and was found to be negative for HLA-B27.

Given the patient’s history, presenting complaints, and investigation findings, a final diagnosis of vitamin E toxicity-related coagulopathy was made. Exogenous vitamin E supplementation was stopped. The patient received intravenous fluids, pantoprazole, vitamin K, vitamin C, multiple transfusions of fresh frozen plasma (FFP), and other supportive treatments.

The patient’s complaints were alleviated with the treatment and the coagulation parameters normalized. The patient was discharged with complete resolution of symptoms and remained asymptomatic during the follow-up for 6 months.

## Discussion

This male patient presented with oral bleeding, melena, and bruising over his back. He was taking NSAIDs infrequently for low backache, and vitamin E for hair loss. Investigations suggested the deficiency of multiple coagulation factors due to acquired vitamin K deficiency. Upper gastrointestinal endoscopy showed gastroduodenal erosions. On the basis of normal serum phylloquinone, increased PIVKA-II levels, and slightly raised serum alpha-tocopherol, the patient was diagnosed as having vitamin E toxicity-related coagulopathy, which responded well to pantoprazole, vitamin K supplementation, fresh frozen plasma transfusions, and other supportive treatment besides the discontinuation of exogenous vitamin E administration. As such, this case documents the inhibition of vitamin K-dependent factors with coagulopathy at only marginally increased levels of serum vitamin E, while vitamin E toxicity-related coagulopathy has been reported earlier at much higher levels of serum vitamin E [5, 8].

The extent of vitamin E excess-induced vitamin K suppression varies as per the dose consumed. Any preexisting subclinical deficiency may be unmasked by any level of vitamin E excess, thereby implying that there may be considerable interpersonal variability reflecting the inhibitory effects of vitamin E on individuals with a poor vitamin K status [9].

Though serum alpha-tocopherol level was only marginally increased and did not confirm the vitamin E-related toxicity convincingly, it is possible that the actual level of vitamin E may be higher than measured, as alpha-tocopherol may not measure the circulating vitamin E accurately.

Vitamin E may compete for the enzyme vitamin K epoxide reductase, which converts the precursors of vitamin K into the active form of vitamin K [3]. As a result, the activation of inactive vitamin K may be inhibited, thereby preventing the gamma-carboxylation, and thus activation, of vitamin K-dependent coagulation factors. The inability to activate the clotting cascade via these factors may increase the risk of bleeding.

Moreover, vitamin E excess decreases the availability of factor IX as it reduces the production of glutamate, which is required for factor IX production [3]. Platelet aggregation is also decreased in patients receiving vitamin E and may be in part due to a mechanism linked to protein kinase C inhibition [10]. The bleeding risk due to a marginally elevated level of vitamin E was also aggravated by NSAIDs in our patient.

Normal serum phylloquinone and increased PIVKA-II levels negate the possibility that this patient had vitamin K deficiency leading to gastrointestinal bleeding/skin changes. Moreover, our subject was a young adult and there was no evidence of any of the causes of vitamin K deficiency in adults, such as malnutrition, lipid malabsorption, malignancy, or renal disease [11].

Prolongation of both PT and APTT, and a normal TT in our patient were suggestive of coagulation factor deficiency in the common pathway (factors II, V, VII, and X), along with factor IX deficiency or presence of inhibitors of prothrombin, fibrinogen, factor V, or factor X. Full correction of the abnormal APTT and PT by pooled normal plasma ruled out factor inhibitors and suggested a deficiency of coagulation factors. The absence of correction of abnormal PT on mixing aged plasma with the patient’s plasma suggested factor V deficiency. The absence of correction of PT on mixing adsorbed plasma with the patient’s plasma was suggestive of factor II, VII, and X deficiency. Moreover, selected assays showed normal factor VIII and raised factor I (fibrinogen) levels, but deficient factor IX levels.

Inherited factor deficiency was not likely because of the absence of any abnormal bleeding until the current episode. Acquired factor deficiency due to liver disease was unlikely in view of normal liver function tests and a normal ultrasound scan of the liver. The absence of thrombocytopenia and a raised serum fibrinogen level excluded the possibility of disseminated intravascular coagulation causing factor deficiency. A raised fibrinogen level may be due to low serum iron concentrations, caused by blood loss, as a negative correlation was observed between total fibrinogen and serum iron concentrations in a large-scale epidemiological study [12].

Anticoagulant-induced factor deficiency was ruled out as the patient was not receiving any anticoagulants. The presence of combined factor deficiencies generally implies severe vitamin K deficiency, but it would be reasonable to assume that a secondary factor must exist, as an isolated vitamin K deficiency is rare due to its continual production by the gut microbiome in healthy individuals [13].

Vitamin E excess potentiated by ill-timed NSAID use could have led to the precipitation of this patient’s complaints [14]. Vitamin E excess could have inhibited vitamin K-related coagulation factor synthesis and prolonged the BT, while NSAIDs could cause gastroduodenal erosions, besides prolonging the BT, and thus add to the risk of gastrointestinal bleeding.

Vitamin E toxicity-induced coagulopathy has been described at high levels of serum alpha-tocopherol, but there is a case report of intracranial hemorrhage attributed to vitamin E toxicity occurring at serum alpha-tocopherol level of 23.3 mg/L [5]. Bleeding manifestations occurred in our case at an even lower level of serum alpha-tocopherol. Our case report highlights the often underscored bleeding risks with vitamin E consumption and that this coagulopathy can also occur at marginally increased levels of vitamin E.

Vitamin E has been lauded for its various dermatological and cardiovascular benefits, all of which are exceptional [15]. However, the same stellar reputation leads to indiscriminate use by prescription, as well as without prescription, by patients. Though the bleeding risk may be hemodynamically relevant in patients already receiving anticoagulants, our case report reveals that the risk of upper gastrointestinal bleeding could also be increased in patients taking both NSAIDs and vitamin E. Moreover, bleeding from gums, ecchymotic patches over the back, normal serum phylloquinone level, and deficiency of multiple coagulation factors, in the presence of increased PIVKA-II level, were highly suggestive of coagulopathy due to marginally increased vitamin E-related inhibition of vitamin K-dependent factors.

 As the patient recovered readily with vitamin K supplementation and FFP transfusions, a final diagnosis of vitamin E toxicity-related coagulopathy was reaffirmed, although NSAID intake might have been an aggravating factor.

## Conclusions

Vitamin E-related inhibition of vitamin K-dependent factors with coagulopathy may occur even at slightly raised levels of vitamin E. This risk is augmented in presence of other drugs that have the potential to cause bleeding.

Thus, patients must be discouraged from taking medications without proper medical consultation. Physicians too must be wary of the potential interactions of substances generally considered harmless.

## Data Availability

Not applicable.

## References

[1] Schmölz L, Birringer M, Lorkowski S, Wallert M (2016). Complexity of vitamin E metabolism. World J Biol Chem.

[2] Dowd P, Zheng ZB (1995). On the mechanism of the anticlotting action of vitamin E quinone. Proc Natl Acad Sci U S A.

[3] Padda IS, Patel P, Citla Sridhar D. Protein S and C. 2022. In: StatPearls. Treasure Island (FL): StatPearls Publishing. https://www.ncbi.nlm.nih.gov/books/NBK557814/. Accessed 21 Sept 2022.

[4] Wypasek E, Undas A (2013). Protein C and protein S deficiency—practical diagnostic issues. Adv Clin Exp Med.

[5] Le NK, Kesayan T, Chang JY, Rose DZ (2020). Cryptogenic intracranial hemorrhagic strokes associated with hypervitaminosis E and acutely elevated α-tocopherol levels. J Stroke Cerebrovasc Dis.

[6] Traber MG (2013). Mechanisms for the prevention of vitamin E excess. J Lipid Res.

[7] Booth SL, Golly I, Sacheck JM, Roubenoff R, Dallal GE, Hamada K (2004). Effect of vitamin E supplementation on vitamin K status in adults with normal coagulation status. Am J Clin Nutr.

[8] Owen KN, Dewald O. Vitamin E toxicity. 2022. In: StatPearls. Treasure Island (FL): StatPearls Publishing; https://www.ncbi.nlm.nih.gov/books/NBK564373/. Accessed 15 Feb 2023.

[9] Capone K, Sentongo T (2019). The ABCs of nutrient deficiencies and toxicities. Pediatr Ann.

[10] Freedman JE, Keaney JF (2001). Vitamin E inhibition of platelet aggregation is independent of antioxidant activity. J Nutr.

[11] Card DJ, Gorska R, Harrington DJ (2020). Laboratory assessment of vitamin K status. J Clin Pathol.

[12] Rautenbach PH, Nienaber-Rousseau C, de Lange-Loots Z, Pieters M (2021). Certain associations between iron biomarkers and total and γ' fibrinogen and plasma clot properties are mediated by fibrinogen genotypes. Front Nutr.

[13] Conly JM, Stein K (1992). The production of menaquinones (vitamin K2) by intestinal bacteria and their role in maintaining coagulation homeostasis. Prog Food Nutr Sci.

[14] Podszun M, Frank J (2014). Vitamin E-drug interactions: molecular basis and clinical relevance. Nutr Res Rev.

[15] Meagher EA (2003). Treatment of atherosclerosis in the new millennium: is there a role for vitamin E?. Prev Cardiol.

